# *Addendum:* Pinto, M.; Gámez, N.; Fuentes, L.; Amor, M.; Horcas, J.M.; Ayala, I. Dynamic Reconfiguration of Security Policies in Wireless Sensor Networks. *Sensors* 2015, *15*, 5251–5280

**DOI:** 10.3390/s150716832

**Published:** 2015-07-13

**Authors:** Mónica Pinto, Nadia Gámez, Lidia Fuentes, Mercedes Amor, José Miguel Horcas, Inmaculada Ayala

**Affiliations:** Departamento de Lenguajes y Ciencias de la Computación, University of Málaga, Andalucía Tech, Málaga 29071, Spain; E-Mails: nadia@lcc.uma.es (N.G.); lff@lcc.uma.es (L.F.); pinilla@lcc.uma.es (M.A.); horcas@lcc.uma.es (J.M.H.); ayala@lcc.uma.es (I.A.)

The authors wish to update the Acknowledgments in their paper published in *Sensors* [1], doi:10.3390/s150305251, website: http://www.mdpi.com/1424-8220/15/3/5251.
